# Causes and timing of recurring subarctic Pacific hypoxia

**DOI:** 10.1126/sciadv.abg2906

**Published:** 2021-06-02

**Authors:** Karla P. Knudson, Ana Christina Ravelo, Ivano W. Aiello, Christina P. Knudson, Michelle K. Drake, Tatsuhiko Sakamoto

**Affiliations:** 1 Department of Earth and Planetary Sciences, University of California, Santa Cruz, 1156 High Street, Santa Cruz, CA 95064, USA.; 2 Department of Ocean Sciences, University of California, Santa Cruz, 1156 High Street, Santa Cruz, CA 95064, USA.; 3 Moss Landing Marine Laboratories, Moss Landing, CA 95039, USA.; 4 Department of Mathematics, University of St. Thomas, 2115 Summit Avenue, St. Paul, MN 55105, USA.; 5 Graduate School and Faculty of Bioresources, Mie University, 1577 Kurimamachiya-cho, Tsu, Mie 514-8507, Japan.

## Abstract

Several North Pacific studies of the last deglaciation show hypoxia throughout the ocean margins and attribute this phenomenon to the effects of abrupt warming and meltwater inputs. Yet, because of the lack of long records spanning multiple glacial cycles and deglaciation events, it is unclear whether deoxygenation was a regular occurrence of warming events and whether deglaciation and/or other conditions promoted hypoxia throughout time. Here, subarctic Pacific laminated sediments from the past 1.2 million years demonstrate that hypoxic events recurred throughout the Pleistocene as episodes of highly productive phytoplankton growth and were generally associated with interglacial climates, high sea levels, and enhanced nitrate utilization—but not with deglaciations. We suggest that hypoxia was typically stimulated by high productivity from iron fertilization facilitated by redox-remobilized iron from flooded continental shelves.

## Teaser

Recurring subarctic Pacific hypoxia is attributed to episodes of high phytoplankton productivity stimulated by iron.

## INTRODUCTION

As a result of human-influenced climate warming and nutrient inputs, dissolved oxygen content in the open ocean has decreased an estimated 2% in the past 50 years, with the greatest reductions observed in the North and Equatorial Pacific (*1*). Whether today’s trend may lead to sudden or extreme hypoxia is not known. Models predict a 1 to 7% decline in global ocean oxygen by the year 2100, with a 1000-year trajectory of diminishing oxygen concentrations, due to warming-induced reductions in gas solubility and convection (*2*, *3*). Yet, models consistently underestimate deoxygenation and cannot replicate observed spatial patterns (*2*), meaning that there are complexities to hypoxia that are still not understood. Paleoceanographic records of deoxygenation events can help us better understand the mechanisms and environmental conditions responsible for oxygen loss, which has been shown to substantially affect biodiversity, food webs and fisheries (*2*, *4*, *5*), ocean acidification (*6*), biogeochemical cycling of important nutrients (e.g., C, N, P, and Fe) (*3*, *6*–*9*), and release of the strong greenhouse gas N_2_O (*2*).

**Fig. 1:**
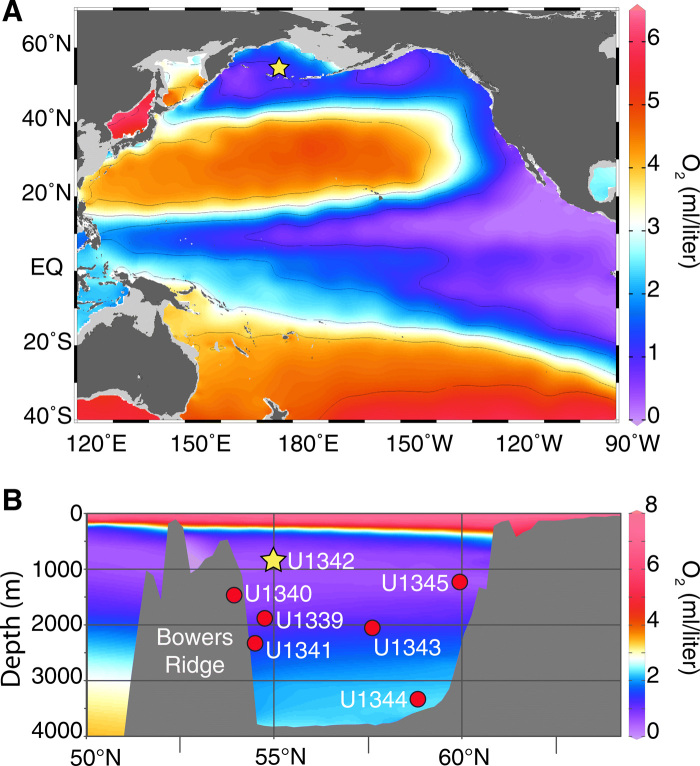
Pacific and Bering Sea dissolved oxygen. (**A**) Map of Pacific dissolved oxygen [O_2_] at 400-m depth, showing pronounced OMZs in the equatorial Pacific and North Pacific margins. EQ, equator. (**B**) Bering Sea profile of dissolved oxygen [O_2_] along the 180°E transect from 50°N to 68°N. Locations of all IODP Expedition 323 Sites are marked. Site U1342 (starred; 818-m water depth), on Bowers Ridge, is located near the modern OMZ, making it an ideal site for detecting changes in oxygenation over time. Figures were generated with Ocean Data View (*65*) using data from World Ocean Atlas 2013 (*66*).

Oxygen minimum zones (OMZs) represent important regions that have unusually low oxygen concentrations ([O_2_] < 0.5 ml/liter) and are particularly susceptible to hypoxia (*7*). OMZs, which are relatively rare in the modern oceans, are typically found in coastal regions with upwelling and high productivity, in enclosed basins, and in areas affected by eutrophication from agricultural runoff (*3*, *10*, *11*). The North Pacific has the largest OMZ area (Fig. 1A) because of weak overturning circulation, O_2_-poor source waters, and high rates of phytoplankton productivity that lead to oxygen-consuming respiration at depth (*3*).

Several marine sediment records from the last glacial-interglacial cycle demonstrate that OMZs across the entire North Pacific intensified during past warming events. Laminated sediments indicate hypoxic conditions in history, because these fine structures can only be preserved if the sediment-water interface dropped past the threshold to sustain bioturbating macrofauna ([O_2_] < 0.1 ml/liter). Lamination preservation is found in sediments from the last deglaciation [ca. 10 to 18 thousand years (ka) ago] at intermediate depths at several Pacific margin locations, including the western California margin (*12*–*15*), the Gulf of California (*16*), the Bering Sea (*17*–*22*), the Gulf of Alaska (*23*), and the Japan margin (*24*, *25*). At these locations, laminations occur ubiquitously during the Bølling-Allerød (B/A; ca. 14.7 to 12.9 ka ago) interstadial and typically (except at the California Margin) during the early Holocene (ca. 11.5 to 10 ka ago).

The laminated B/A event has been largely attributed to increased phytoplankton productivity (*18*, *19*, *23*, *26*–*28*)—although changes in the pathway and composition of North Pacific Intermediate Water (NPIW) (*12*–*15*, *28*, *29*), and a combination of both mechanisms (*17*, *30*), have been suggested. Enhanced primary productivity during these hypoxic events has been linked to the effects of abrupt warming (*9*) and meltwater pulses from Northern Hemisphere ice sheets (*21*, *23*, *31*–*33*), with remobilization of iron from continental shelves serving as a positive feedback (*9*, *23*). However, there are limited studies directly testing the iron fertilization mechanism during these deglacial events, and the findings do not agree (*31*, *34*).

While the last deglaciation has been extensively studied, no previous records from North Pacific OMZ regions have extended beyond the last glacial-interglacial cycle. Hence, it remains speculative how to use our knowledge of the last deglacial’s hypoxic events to inform our understanding of ocean conditions under other time periods—past or future. For example, there is strong evidence that Pacific meltwater fluxes during the deglaciation triggered abrupt climatic and oceanic changes with possible global impacts (*33*). Whether this forcing mechanism is the primary trigger for North Pacific hypoxic events is an idea that the paleoceanographic community cannot begin to test without a long record containing many occurrences of hypoxia.

To this end, we generated extensive regional paleoceanographic records over the past 1.2 million years (Ma), which provide a much needed broader context that goes beyond the wealth of existing deglacial studies. Specifically, the aim of our work is to determine (i) whether hypoxia is a regular feature of most or all deglacial warming transitions, (ii) whether the mechanisms underlying hypoxia are consistent throughout time, and (iii) whether the susceptibility of margin environments to the development of hypoxia depends on certain background conditions—including Milankovitch climate cycles, sea level, ocean stratification, and ocean ventilation.

### Subarctic Pacific sensitivity to deoxygenation

**Fig. 2:**
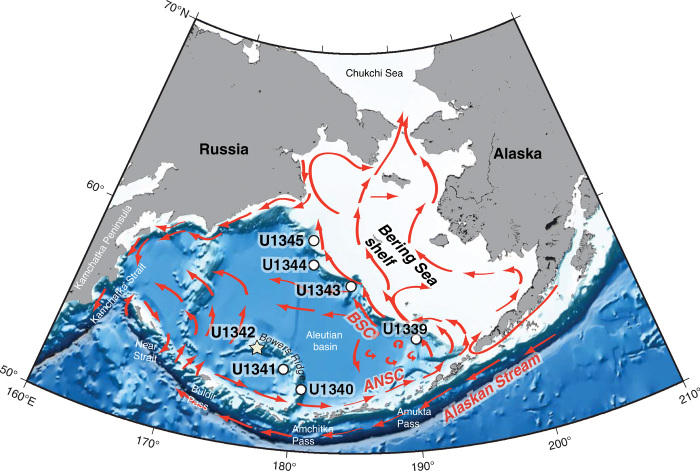
Bering Sea surface circulation map. IODP Expedition 323 Sites within the Bering Sea, including Site U1342 on Bowers Ridge (starred). Red arrows indicate directions of surface currents. The Bering Sea exchanges water with the North Pacific through the Aleutian Island Arc via the Kamchatka Strait (4000-m sill depth), Near Strait (2000-m sill depth), and numerous other smaller passages, allowing the Bering Sea to record changes in North Pacific intermediate and deep water conditions (*44*). The Alaskan Stream, the greatest source of water flow into the Bering Sea, enters the basin through the Aleutian Arc and flows eastward, forming the Aleutian North Slope Current (ANSC) (*44*). The ANSC turns northwestward near the continental shelf, forming the Bering Slope Current (BSC), the primary current responsible for advection from and across the continental shelves (*44*). Modified from IODP Expedition 323 Preliminary Report (*38*).

The Bering Sea, one of the world’s most thriving and diverse ecosystems (*35*), is extremely vulnerable to changes in oxygenation. The Bering Sea presently experiences sluggish circulation and weak ventilation—although during past glacials, NPIW was enhanced because of locally formed brine rejection (*36*). Exceptionally high productivity and strong stratification further promote low-oxygen conditions and contribute to a seasonal OMZ (*10*, *11*), which represents 7% of Earth’s total OMZ surface area (*11*). Integrated Ocean Drilling Program (IODP) Site U1342 (818-m water depth) on Bowers Ridge in the Bering Sea (Figs. 1 and 2) is an episodically laminated site ideally located to detect subarctic Pacific dissolved oxygen variability. The site is located just above the modern OMZ and records glacial-interglacial variability in NPIW ventilation (*36*).

This study offers an unprecedented examination of the environmental conditions underlying North Pacific hypoxic events over many climate cycles (0 to 1.2 Ma ago), using high-resolution geochemical and sedimentological records from Site U1342, in addition to Monte Carlo hypothesis testing. This work provides the first evidence that hypoxic events recurred throughout the Pleistocene as high-productivity episodes and were generally associated with interglacial climates and high sea levels but not typically with deglaciations. Moreover, this study shows that enhanced nitrate utilization and possible changes in iron source regularly coincided with hypoxic events, while changes in ventilation may have only occasionally or weakly influenced hypoxia. Synthesizing this evidence, we suggest that high interglacial sea levels provided an important background condition for recurring hypoxia, in which redox-remobilized iron from flooded continental shelves enabled high productivity to be triggered by events that carried an influx of nutrients into the photic zone (e.g., strong upwelling or altered surface currents).

### Bering Sea laminated sediments record hypoxia

In the next sections, we evaluate to what extent deoxygenation events, recorded as preserved laminated sediments, are linked to several possible contributing environmental factors—including changes in climate, primary productivity, sea level, NPIW ventilation, stratification, and iron source. It is worthwhile to note that this study identifies hypoxia at Site U1342 on the basis of the preservation of grouped laminations and other fine structures, and it is not focused on the original sedimentation and individual laminae.

The nature and resolution of Bering Sea laminated sediments is still somewhat unresolved and likely differs by location within the basin, prohibiting direct comparison of different sites at a laminar scale. Many previous Bering Sea studies lack sufficient age control to definitively determine whether layers have annual or other consistent variation (*17*, *18*, *20*). Detailed x-ray fluorescence (XRF) investigations of laminated sediments from the Bering Sea northern slope during the last deglaciation appear to demonstrate annual varves (*19*), although work from other Bering Sea locations has hypothesized that layers are not annual (*20*).

At the top of Bowers Ridge, where Site U1342 is located, winnowing and occasional cross-bedding complicate the sedimentation structures (*37*), and the relatively low sedimentation rate (4.5 cm/ka) compared to other Bering Sea locations (12 cm/ka at other Bowers Ridge sites and 28 to 45 cm/ka on the Bering Slope and Umnak Plateau) makes individual laminae less prominent. However, for the purposes of this study, distinguishing the exact nature of sedimentation at Site U1342 is not critical, because we focus on the mechanisms that contribute to hypoxia—which allows the laminations and other fine structures (e.g., cross-bedding) to be preserved—rather than the specific processes that originally create the layered sediments.

## RESULTS AND DISCUSSION

### Hypoxic events typical of interglacial climates

**Fig. 3:**
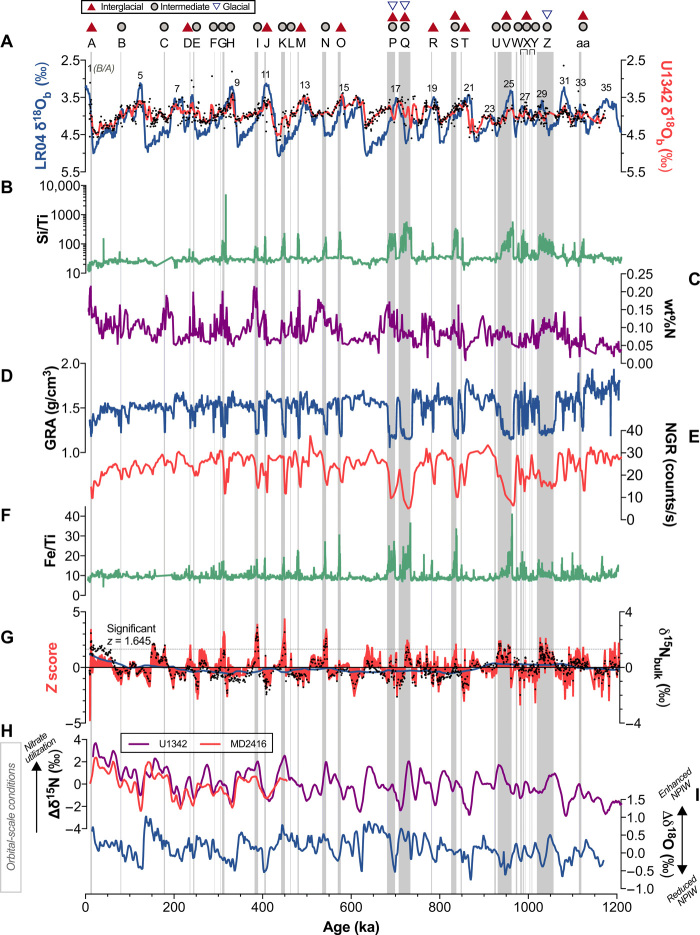
Occurrences of laminated sediments at Site U1342 compared to global and regional records of climate and environmental changes. Preserved laminated intervals (gray vertical bars), indicating low-oxygen conditions ([O_2_] < 0.1 ml/liter), compared to (**A**) benthic δ^18^O from the LR04 stack (*53*) (blue line) representing global climate, in which larger values are more glacial, and benthic δ^18^O from Site U1342 (*36*) reflecting regional climate and NPIW presence (black dots are raw data and red line indicates smoothed average). The climate type(s) associated with each laminated interval (assigned on the basis of U1342 benthic δ^18^O; see Materials and Methods) are shown above the lamination name (interglacials, solid red triangles; intermediate climates, gray circles; glacials, blue open triangles). Several records indicate that preserved laminations represent high-productivity events, including (**B**) Si/Ti ratios calculated from XRF analyses at Site U1342, in which high values indicate enhanced biogenic opal deposition; (**C**) weight % nitrogen at Site U1342 (*45*), in which high values indicate enhanced organic matter preservation; (**D**) bulk density measured by gamma ray attenuation (GRA) at Site U1342, in which relatively low values reflect high percentages of biogenic opal and whole-diatom preservation (*38*); and (**E**) natural gamma ray (NGR) spectra at Site U1342 (*38*), in which low values indicate high biological sediment composition and decreased clay content. Laminated intervals also correlate to peaks in (**F**) Fe/Ti at U1342, which may indicate a change in iron source. In (**G**), calculated *z* scores (red) of Site U1342 bulk sedimentary δ^15^N measurements (black dots) show that significant peaks in the δ^15^N record are overall correlated with laminated intervals; δ^15^N running mean used to calculate *z* scores is shown as a navy line. (**H**) Nitrate utilization proxies (∆δ^15^N) at Site U1342 (*45*) (Site U1342 δ^15^N minus Site 1012 δ^15^N; purple line) and Site MD2416 (*67*) (Site MD2416 δ^15^N minus Site 1012 δ^15^N; red line), and (**I**) NPIW proxy (∆δ^18^O) (*36*) show orbital-scale background conditions.

**Fig. 4:**
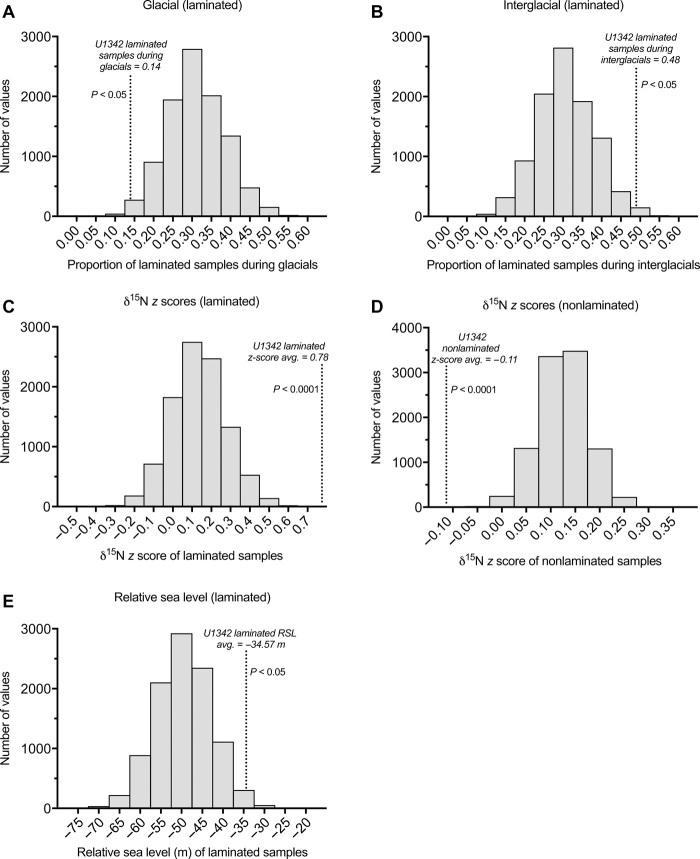
Monte Carlo hypothesis test results. In all, histograms show randomized test statistics simulated by Monte Carlo techniques, and dotted lines indicate results from original sample data. Distinct differences between randomized distributions and original data, indicating significant findings (*P* < 0.05), are shown in (**A**) the proportion of laminated samples during glacial climates, (**B**) the proportion of laminated samples during interglacial climates, (**C**) δ^15^N *z* scores of laminated samples, (**D**) δ^15^N *z* scores of nonlaminated samples, and (**E**) mean relative sea level associated with laminated samples.

The temporal occurrences of laminated sediments were examined to determine whether hypoxia was regularly linked to any climate regimes. Site U1342 contains 27 intervals of laminated sediments, which do not occur at consistent time intervals and range from punctuated events shorter than 1 ka to long-lived hypoxic “states” persisting up to nearly 40 ka (Fig. 3 and tables S1 and S2). Laminated intervals were assigned climate types based on corresponding U1342 benthic δ^18^O (see Materials and Methods). Nearly all (24 of the 27) of the laminated intervals were associated with only interglacial and/or intermediate climates, and no laminated intervals were exclusively associated with a glacial climate. Three laminated intervals spanned glacial climates as well as intermediate and/or interglacial climates. Monte Carlo hypothesis tests confirm that U1342 laminated intervals did not occur randomly but were significantly associated with interglacial climates (*P* ≤ 0.05) and were unlikely to occur during glacial climates (*P* ≤ 0.05) (Fig. 4, A and B).

Laminated sediments were not consistently linked to deglaciations, serving as important evidence that tests whether deglacial mechanisms typically trigger hypoxia. Like many other North Pacific margin records, Site U1342 displays well-preserved, notable laminated sediments at the B/A (although the laminations are complicated by cross-bedding, prohibiting direct laminae-scale comparison to other high-resolution records; see table S2). Before the last glacial cycle, the only other occurrences of laminated sediments confined strictly to deglaciations were at marine isotope stage (MIS) 7 to 8 (interval “E”), MIS 13c to 14 (interval “N”), and MIS 23 to 24 (interval “U”).

In summary, while a few exceptions exist, subarctic Pacific hypoxic events were primarily recurring features of interglacial and intermediate climates—but did not regularly occur as distinctive deglacial events. Thus, the meltwater pulses characteristic of the deglaciations are unlikely to explain most deoxygenation events. It is theoretically possible that abrupt warming could occur during other background states besides deglaciations, but no long regional temperature records exist to investigate this idea. Therefore, we cannot rule out the possibility that abrupt interglacial warmings could serve as a trigger for a hypoxic event. Yet, the unprecedented findings strongly linking hypoxia and interglacial climates warrant a thorough exploration of possible causes; this is the aim of our proceeding analyses.

### High productivity linked to hypoxia

**Fig. 5:**
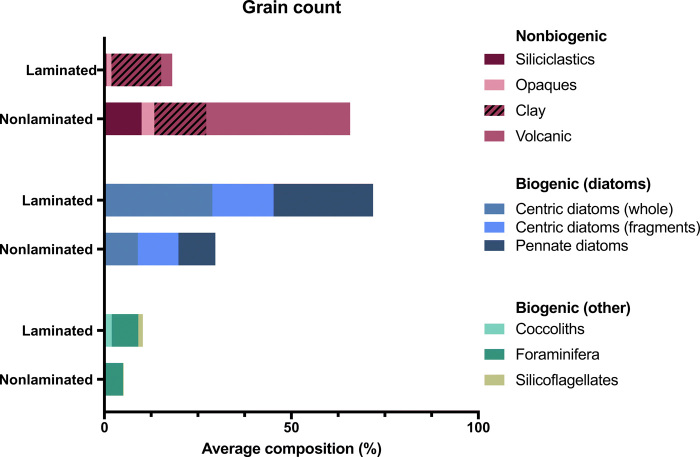
Smear slide grain counts from Site U1342. Counts show distinct differences in biogenic and nonbiogenic compositions between laminated and nonlaminated sediments, which indicate that laminated sediments reflect high-productivity events.

Several independent parameters measured on cores from Site U1342 show that laminated sediments are characterized by high productivity, indicating that enhanced productivity and subsequent enhanced organic matter export and subsurface oxygen utilization played primary roles generating hypoxia throughout the Pleistocene (see Materials and Methods for proxy descriptions and justification.) Smear slide analyses show that laminated sediments contain substantially more diatomaceous biogenic material relative to siliciclastic grains (Fig. 5). Laminated samples contain a higher percentage of total diatoms compared to nonlaminated samples (72 versus 30%) and a higher percentage of well-preserved (i.e., whole, nonfragmented) diatoms (29 versus 9%), indicative of a high siliceous flux rate. Laminated sediments also display higher proportions of other biogenic particles (including silicoflagellates, sponge spicules, foraminifera, and coccolithophores). The fact that the assemblages are always dominated by diatoms, rather than other microfossil groups, suggests that Pleistocene oceanographic and nutrient conditions were probably within the range that occurs in the modern Bering Sea, with gradients in diatom productivity influenced by iron and nutrient supply from the continental shelf (*35*). A lower abundance of volcanic grains during laminated (3%) compared to nonlaminated (39%) intervals may reflect an increase in diatomaceous material during laminated intervals that diluted the volcanic contribution, as there are no known mechanisms that would result in reduced volcanic production during laminated intervals.

In addition, Si/Ti (a proxy for the presence of biogenic opal), weight percent (wt%) N (used to indicate the relative presence of preserved organic matter), and bulk density measured by gamma ray attenuation (GRA) all demonstrate significantly high primary productivity coincident with laminated intervals (Fig. 3 and fig. S1). Laminated and nonlaminated sediment values for Si/Ti and wt% N are 102.78 versus 30.71 (*P* < 0.0001) and 0.10 versus 0.08 (*P* < 0.01), respectively. GRA is significantly lower during laminated intervals compared to nonlaminated intervals (1.25 g/cm^3^ versus 1.59 g/cm^3^, *P* ≤ 0.0001), primarily because laminations contain higher percentages of whole diatoms (which provide greater pore space than diatom fragments), and secondarily because laminations are characterized by low-density biogenic opal (*38*). Natural gamma radiation (NGR) is significantly lower during laminated intervals compared to nonlaminated intervals (14.68 counts/s versus 26.25 counts/s, *P* ≤ 0.0001), showing a lithological change to decreased clay content (*39*) during laminations. Other long, well-dated, high-resolution records of subarctic Pacific productivity are lacking for comparison. However, the finding that laminated sediments represent high-productivity events agrees with deglacial (B/A) (*18*–*20*, *22*, *40*, *41*) and Pre-Boreal (ca. 11 to 12 ka ago) (*19*, *20*, *41*) records from the Bering Sea and other North Pacific locations. A detailed examination from 0 to 275 ka ago of two other Site U1342 productivity records, wt% C and carbon mass accumulation rate, also indicates that productivity was high during laminated intervals (*36*).

### Hypoxia in the context of ventilation conditions

Now that the link between productivity and hypoxia has been presented, we must consider whether hypoxic events may also be related to changes in subsurface ocean ventilation from oxygen-rich surface waters on rapid and/or glacial-interglacial time scales. High-resolution studies of Bering Sea ventilation over the last deglaciation using benthic minus planktonic foraminifera radiocarbon ages show no remarkable variation between laminated and nonlaminated intervals at nearby IODP Site U1340 (1324 m) on Bowers Ridge (*18*). Two sites off of the northern Bering Sea shelf also observe no notable deglacial ventilation changes (*19*), while a site from the western Bering Sea records reduced ventilation during the B/A (*42*). Thus, studies from the last deglaciation suggest that high-frequency changes in NPIW ventilation or circulation were not necessarily required to enable hypoxia at Bowers Ridge and perhaps exerted variable influences at more remote Bering Sea locations.

Site U1342 is the sole source of information on possible high-frequency changes in ventilation that occurred before the last deglaciation. Oxygen isotope records from this site demonstrate that rapid reductions in ventilation only occasionally assisted development of hypoxia. The surface waters of the Bering Sea are characterized by very negative δ^18^O, which transmit their signal to depth during periods of enhanced brine formation and NPIW ventilation (*36*). At Site U1342, we observe that some interglacial and intermediate climate laminated intervals (most notably interval “J” at MIS 11, as well as intervals “H,” “P,” and “S”) correspond to brief uncharacteristically high benthic δ^18^O values (as high as ~5‰ at MIS 11; Fig. 3A), which likely reflect reduced NPIW ventilation and relatively diminished influence of very negative δ^18^O Bering Sea surface waters (*36*). However, most U1342 laminated intervals do not show a δ^18^O excursion attributed with enhanced ventilation. Overall, hypoxia is much more consistently linked throughout time to high productivity than to rapid NPIW ventilation changes.

While high-frequency or rapid ventilation changes were not essential for driving hypoxic events, ventilation changes on broad glacial-interglacial time scales may have provided background conditions that made productivity-driven hypoxic events more likely. Site U1342 yielded the longest available high-resolution records of orbital-scale NPIW variability. These records show that NPIW ventilation was diminished during interglacial/weak glacials and enhanced during strong glacials because of localized brine formation within coastal polynyas (*36*). Consistent with this, NPIW proxy records that represent the difference in δ^18^O between the intermediate-depth Site U1342 and the deep Pacific (*36*) (∆δ^18^O; Fig. 3I) show that ventilation was diminished during laminated intervals compared to nonlaminated intervals (0.056‰ versus 0.160‰). Because this result is not strongly significant (*P* = 0.115), it suggests that—to some limited extent—weak interglacial ventilation may have preconditioned the region to be susceptible to hypoxia, while high productivity and subsurface organic matter respiration primarily drove the hypoxic events.

In addition, laminated intervals at U1342 tend to be thicker during the Mid-Pleistocene Transition (MPT; ~1200 to 700 ka ago) compared to after the MPT (Fig. 3), and this may also be explained by a long-term background change in ventilation. The MPT is characterized by a fundamental shift in dominant glacial-interglacial cycles from 40 to 100 ka, and this change was associated with reduced upwelling, expanding sea ice extent, and enhanced NPIW formation and ventilation, which may have been attributed to the more frequent closure of the Bering Strait (*36*, *43*). Therefore, there appears to be a ventilation change over time toward conditions less favorable for hypoxia, but high-productivity events—particularly during interglacials—were still capable of overcoming these conditions.

### Evidence for iron fertilization linked to high productivity and hypoxia

The Bering Sea is one of the world’s most productive regions, particularly along the continental shelf “green belt,” where nutrient-rich waters are advected into the photic zone (*35*). Nutrient advection is strongly affected by the Bering Slope Current (BSC), an extension of the relatively warm Alaskan Stream, which enters the basin though the Aleutian Islands (*44*) and contributes to the surface gyre (Fig. 2). Presently, productivity in much of the Bering Sea is iron-limited, particularly at sites (such as U1342) (*45*) that are more distal from the continental shelf, which represents an important source of iron to the basin (*35*, *46*).

In iron-limited surface waters, enhanced net primary productivity can occur from two main scenarios: (i) the addition of external iron to the surface waters (i.e., “iron fertilization”), such that the surface nitrate pool can be used more completely (recorded by enhanced δ^15^N_bulk_), and/or (ii) vigorous upwelling of nutrient-rich waters. Upwelled waters are accompanied by some iron, but the ratio of iron to major nutrients is not high enough to relieve iron limitation and thereby substantially fractionate the surface nitrate pool (δ^15^N_bulk_ values are not enhanced). However, an “iron fertilization/upwelling” combination scenario would yield high values of δ^15^N_bulk_, because increased iron abundance would promote utilization of the additional nutrients.

To distinguish between the possible productivity mechanisms associated with laminated sediments, we analyzed the Site U1342 δ^15^N_bulk_ record from Knudson and Ravelo (*45*) in coordination with productivity records from the same site (see Materials and Methods for justification of δ^15^N_bulk_ measurements at this site). *Z* scores were calculated to determine statistically significant peaks in δ^15^N_bulk_. *Z* scores were significantly higher during laminated intervals compared to nonlaminated intervals (0.779 versus −0.112, *P* ≤ 0.0001; Fig. 3G and table S1). Monte Carlo hypothesis tests also show a strong association between high *z* scores and laminated samples (*P* < 0.0001; Fig. 4, C and D), indicating that high productivity was strongly associated with peaks in nitrate utilization, consistent with the iron fertilization scenario and/or the iron fertilization/upwelling combination scenario. A few exceptions, which did not have significantly high *z* scores (brief laminated intervals “J,” “L,” “O,” and “P”), may reflect upwelling without iron fertilization or limitations of the *z*-score calculations over short time windows (see Materials and Methods). Overall, the results strongly link high productivity, found during laminated intervals, with enhanced nitrate utilization and iron fertilization.

While the Site U1342 δ^15^N_bulk_ record may also partially reflect water column denitrification and/or changes in the δ^15^N of source nitrate (*45*) (also see Materials and Methods), we assert that these processes would accompany changes in productivity and therefore may amplify—but not independently drive—δ^15^N_bulk_ peaks. Water column denitrification, which does not occur in the region today, requires nearly anoxic conditions. Thus, denitrification must result from productivity-induced hypoxia, because there is no consistent evidence for high-frequency changes in ventilation associated with laminations. In addition, an increase in source water δ^15^N alone could not account for peaks in recorded δ^15^N_bulk_ during the highly biogenic, laminated intervals, unless the foreign source water also caused an increase in productivity (e.g., by bringing additional sources of nutrients and/or iron). Therefore, the possibility that enhanced denitrification amplified the δ^15^N signal once hypoxia was initiated does not interfere with our overall interpretations that relative changes in δ^15^N_bulk_ can be interpreted in the context of productivity and nitrate utilization.

### Weak physical ocean stratification promoting hypoxia during interglacials

The Site U1342 δ^15^N record may also be used to discern whether there is a relationship between hypoxia and physical ocean stratification, one of the mechanisms invoked for the last deglaciation (*9*, *31*). This idea can be tested because strong stratification enables more complete nitrate utilization (high δ^15^N_bulk_) but does not typically result in high net productivity, because of the limitation of nutrients in the photic zone. We find that high-productivity, laminated events occur in association with δ^15^N_bulk_ peaks and thereby reject the idea that enhanced stratification regularly promoted hypoxia at our site. We consider strong stratification to be a possible cause of high productivity and laminated sediments only in the rare occasions of deglacial hypoxia, through unique deglacial mechanisms, such as proposed by previous studies (*9*, *31*).

In most cases, we suggest that relatively weak stratification, which occurs in the region during interglacials (when most of the laminated events are observed), may have provided an important background condition promoting productivity and hypoxia. During interglacials, physical stratification relaxes (*45*) and enables deep upwelling to replenish nutrients in the photic zone. In contrast, cool glacial conditions create a strongly stratified water column in the ocean overall (*45*), even with enhanced glacial brine formation and NPIW ventilation occurring in very localized areas within coastal polynyas (*36*).

### Mechanisms for iron-stimulated productivity and hypoxia

**Fig. 6:**
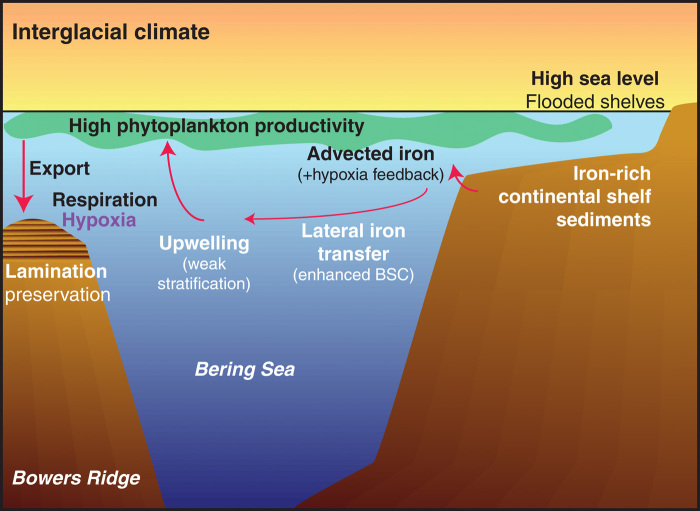
Schematic diagram of processes leading to high productivity and hypoxia. During interglacial and intermediate climates, relatively high sea levels flood continental shelves, allowing iron to be advected into the upper water column. High sea levels also contribute to a strengthened BSC, which promotes lateral advection of iron into the open basin. Weak interglacial stratification (*45*) enables strong upwelling, bringing new sources of iron and nutrients into the photic zone, stimulating phytoplankton productivity, and resulting in enhanced nitrate utilization. High amounts of organic matter are then exported and result in high respiration rates, leading to an expanded OMZ and hypoxia at intermediate depths, including Bowers Ridge Site U1342. Thus, preserved laminated sediments at Site U1342 reflect low-oxygen conditions, high productivity, and enhanced nitrate utilization and correlate to relatively high sea levels.

Some previous studies have suggested that lateral transport of iron from continental shelves to the open ocean stimulated high-productivity hypoxic events in the North Pacific margins during the last deglaciation, due to rising sea levels (*23*) and rapid increases in temperature that may have caused a shoaling of the hypoxic boundary (*9*). A related iron fertilization mechanism may have been active in the subarctic Pacific during interglacial and intermediate climates, when preserved laminations were most prevalent (Fig. 6). During these climate regimes, relatively high sea levels covered continental shelves that could have provided a distal source of iron, whereas low sea level stands associated with strong glacials would have cut off this source. In addition, high sea levels strengthen the BSC, as more Alaskan Stream surface water flows from the North Pacific through the Aleutian Island Arc into the Bering Sea, creating a strong nutrient delivery system to the open basin (*21*, *44*).

Monte Carlo hypothesis tests show a clear, significant link between laminated intervals and high global relative sea level (RSL) (−35 m during laminated intervals versus −51 m during nonlaminated intervals, *P* < 0.05; Fig. 4E), supporting the idea that flooded continental shelves may have fostered favorable conditions for hypoxia. While regional sea levels are difficult to constrain precisely, we are confident that regional sea levels during interglacials were higher than during glacial periods (*47*, *48*) and high regional sea levels would have occurred regardless of whether the major melted ice sheets were in the Northern or Southern Hemisphere (*49*). With the exception of the well-studied B/A and three other events, laminated events did not occur during deglacial periods and avoided associated sea level complications attributed to postglacial isostatic rebound.

In addition, new high-resolution XRF measurements of Fe/Ti at U1342 were used to look at possible changes in iron source (Fig. 3F). Significantly high Fe/Ti ratios during laminated intervals compared to nonlaminated intervals (12.567 versus 9.179, *P* ≤ 0.001) lend support to the idea that iron fertilization from the continental shelf promoted high productivity during hypoxic events, although there are potential caveats (Supplementary text S1). This pattern would not be expected from a dust iron source. The Fe/Ti ratio would change only slightly with dust deposition, because Ti is present in dust and not in other iron sources. Furthermore, no other iron source besides continental shelves could explain the strong link between productivity and interglacial/intermediate climates, as there is no evidence for enhanced interglacial flux of Asian dust (*50*) (i.e., the largest dust source to the subarctic Pacific), enhanced interglacial volcanism, or enhanced interglacial riverine input of iron-bearing minerals to the Bering Sea (*51*).

It is important to make the distinction that it does not appear that sea level rise itself was the initial “trigger” for hypoxia in most cases. Hypoxic events were not initiated once sea level crossed any certain threshold; instead, they occur as punctuated events within periods of high sea level stands. Thus, it is likely that high sea levels provided an important background condition that helped to relieve iron limitation in the region but that other events typically acted as the trigger. In the open subarctic Pacific, micrometer-sized Fe particles in the upper 200 m can be laterally carried more than 800 km from shore (*46*). As a marginal sea, the Bering Sea is surrounded with continental shelves close enough to have provided particulate iron to the site. Wind-driven upwelling (especially promoted by interglacial weak stratification) would have enabled iron to enter the photic zone, thereby fostering high rates of biological productivity. This iron delivery mechanism would be capable of switching on and off through changes in the strength or direction of Bering Sea surface currents (particularly the BSC) and/or upwelling (in part modified by sea ice coverage and wind strength and direction). Thus, high sea levels allowed iron on the continental shelves to be generally accessible, while fluctuating forces in the iron and nutrient delivery system could have acted as triggers of productivity and hypoxia.

Once hypoxia was initiated, enhanced iron remobilization of the shelf sediments could provide a positive feedback on productivity and deoxygenation (*9*, *23*). However, as the conditions progressed, water column denitrification would work to convert biologically usable nitrate into gaseous nitrogen (N_2_ and N_2_O), which would be lost to the atmosphere. This nutrient loss could create a negative feedback that would decelerate productivity and decrease nitrate utilization and allow oxygen levels to eventually be restored.

### Understanding Pacific-wide hypoxia throughout time

The B/A deglacial event, which represents the most recent and most well-documented hypoxic event in the North Pacific, is commonly used as an analog for future hypoxic events predicted to accompany global climate warming. During the last deglaciation, hypoxia was likely triggered by abrupt warming, meltwater pulses from Northern Hemisphere ice sheets, and/or sea level change (*9*, *21*, *23*, *31*–*33*), and this might be expected for other deglacial hypoxia events throughout the Pleistocene. However, here, we show that hypoxia most commonly occurs during warm interglacial stages, rather than being a unique, characteristic feature of deglaciations. Thus, we find that deglacial transitions (and associated meltwater and sea level rise) do not always drive hypoxia, and there is no evidence of a unifying trigger mechanism for hypoxic events throughout time.

While a universal trigger may not exist, our results do highlight key interglacial background conditions that likely primed the region for hypoxia. This study shows that hypoxic events throughout the Pleistocene were linked to high productivity, high sea levels, enhanced nitrate utilization, and feasible iron source changes. We adopt the idea that high sea levels enabled access to iron sourced from continental shelves, which relieved nutrient limitation in the source waters and enabled high productivity that resulted in hypoxia. A related mechanism has been suggested for the last deglacial (*9*, *23*) but never before demonstrated to occur regularly on longer time scales. We newly assert that iron fertilization mechanisms typically occurred during intervals with a background state of relatively high sea level (but are not necessarily triggered by sea level rise) and reduced stratification (enabling enhanced upwelling) and that deglacial transitions do not provide the necessary conditions for all hypoxic events.

To date, the regional studies that have attempted to directly test the iron fertilization mechanism have been limited, are in disagreement with each other, and do not look beyond the last deglaciation (*31*, *34*). Thus, our work offers an advancement to the current understanding of hypoxia and the role of iron in the North Pacific by (i) contributing a rare test for the iron fertilization mechanism, which is based on geochemical and statistical analyses; (ii) providing the first evidence for a fairly consistent mechanism for hypoxia over long time scales; (iii) describing a mechanism that is applicable to situations independent of deglacial conditions; and (iv) identifying important background conditions (i.e., high sea level, weak stratification, and possibly weak ventilation) that promote the development of hypoxia throughout the Pleistocene. Given the potential importance of iron in the North Pacific, these findings emphasize the need to incorporate iron recycling and positive/negative productivity-hypoxia feedbacks into biogeochemical models, which so far focus on changes in gas solubility, ocean circulation, and convection (*52*).

More work must also be done to better understand the hypoxia triggers that took advantage of the iron availability during interglacial sea level high stands. During interglacials, high productivity driven by enhanced upwelling (facilitated by reduced interglacial stratification), and/or changes in the strength and direction of the BSC (directly influenced by high sea level) could have triggered hypoxic events. High-resolution records, such as of biological assemblages, may be able to discern these changes. It is also possible that abrupt warming events within interglacials and subsequent reductions in O_2_ solubility could have driven hypoxic events, which could then be reinforced by an iron fertilization-productivity feedback as suggested for the last deglaciation (*9*, *23*). Presently, no long regional temperature records exist to investigate this idea. Hence, we recommend future work to reconstruct long, high-resolution temperature records that may be suitable for evaluating whether there is a recurring regional link between abrupt warming and hypoxia. Last, more long, high-resolution records of laminated sediments from other geographic regions and depths are necessary to confirm that high productivity is a characteristic feature of basin-wide hypoxic events over long time scales. Interglacial tendencies for productivity and hypoxia over a Pacific-wide scale would be strong support for a simple mechanism of iron delivery such as the one that we propose here, whereas separate regional trends would imply individualized mechanisms to promote hypoxia.

## MATERIALS AND METHODS

### Experimental design

The major objectives of this work are as follows: (i) to determine the occurrences of preserved laminated sediments (recording hypoxia) from Bering Sea IODP Site U1342 over the past 1.2 Ma to evaluate whether they were regularly associated with any particular climate regimes; (ii) to determine whether there was a consistent relationship between productivity and laminated sediments throughout time; (iii) to test whether rapid peaks in productivity characteristic of hypoxic events were typically attributed to iron fertilization, upwelling, or a combination of both mechanisms; and (iv) to determine whether the susceptibility of margin environments to the development of hypoxia depended on certain background conditions—including Milankovitch climate cycles, sea level, ocean stratification, and ocean ventilation. As described in the following sections, this work uses long, multiproxy geochemical records of productivity, ventilation, nitrate utilization, and elemental ratios in combination with robust Monte Carlo hypothesis testing to investigate these objectives.

### Site U1342 sediment cores and laminated intervals

Cores from IODP Site U1342 Holes A, C, and D were sampled at ~3-cm intervals within massive sediments and 1-cm intervals within laminated sediments, yielding an average sampling frequency of ~1 ka. Here, we use the U1342 age model from Knudson and Ravelo (*36*), which was assigned on the basis of visual correlation of benthic foraminiferal δ^18^O to the LR04 benthic δ^18^O stack (*53*). Sediments were characterized for the presence/absence of laminations based on visual inspection of core sediments and photos. Intervals containing the presence of laminations or layers (submillimeter to multiple-centimeter in scale), including partially bioturbated laminations and layers, are referred to as “laminated intervals” and assigned an alphabetical label for reference purposes.

### Climate type(s) of laminated intervals

Laminated samples were defined as “Glacial” or “Interglacial” if they were 1σ above or below a 100-ka moving window of an interpolated three-point running mean of U1342 benthic foraminiferal δ^18^O from Knudson and Ravelo (*36*). All other samples were defined as being associated with “Intermediate” climates. Some relatively long laminated intervals may be associated with more than one climate regime.

### X-ray fluorescence

We present new records of elemental ratios from U1342 sediment cores measured by nondisruptive XRF with the TATSCAN-F2 at the Japan Agency for Marine-Earth Technology. This instrument, analytical methods, precision, and accuracy are described in detail by Sakamoto *et al.* (*54*). Si/Ti, Fe/Ti, and Fe/S ratios were calculated on the basis of the mass measurements (at 0.50-cm resolution) of each of these elements. XRF measurements are likely not greatly affected by water attenuation, because precautions were taken to scan IODP cores after they had been opened and were no longer damp. Core tops (including the B/A laminated interval at this site) may be an exception for XRF reliability, as they are relatively soft and have greatest porosity and water content.

### Smear slide (grain count) and particle size analyses

New records of sediment composition from laminated and nonlaminated were determined from smear slide (quantitative grain counts) and particle size analyses. A subset of samples from three laminated intervals (672 to 697, 826 to 836, and 847 to 851 ka ago) and four adjacent massive intervals (668 to 671, 822 to 825, 803 to 845, and 853 to 856 ka ago) were selected for analyses. Sediment smear slides were examined with a transmitted light petrographic microscope equipped with a standard eyepiece micrometer. Biogenic (pennate and centric diatoms, silicoflagellates, sponge spicules, foraminifers, and coccolithophores), mineral (clay minerals, silt- to sand-size siliciclastics), and volcaniclastic components were identified, and their percentage abundances were visually determined under a petrographic microscope using a 40× objective and 10× eyepiece [e.g., Rothwell (*55*)]. For each sample, three counts were done on different parts of a smear slide using a random walk, and the average value of the three counts was used.

Particle size analyses on the same samples were carried out with a Beckman-Coulter LS 13 320 laser particle size analyzer attached to an aqueous module equipped with a pump and a built-in ultrasound unit. Size distributions were analyzed from 0.04 μm to 2 mm. Measurements of such a wide particle size range are possible because the particle sizer is composed of two units: a laser beam for conventional (Fraunhofer) diffraction (from 0.4 μm to 2 mm) and a polarized intensity differential scatter (PIDS) unit, which measures particles on the basis of the Mie theory of light scattering (0.04 μm) (*56*). The samples for the analyses were subsampled and dispersed in the deionized water of the aqueous module of the particle sizer until obscuration values of 10 to 15% and PIDS obscuration values of 48 to 52% were obtained. The optical model chosen for the grain size determination is the default Fraunhofer model, based on the Fraunhofer theory of light scattering. Data interpolation and statistical analyses were calculated with the laser particle sizer proprietary software (*56*). Because all samples analyzed tend to log-normal grain size distributions in the 0.04-μm to 2-mm spectrum, geometric rather than arithmetic statistics were applied to the values obtained by the logarithmically spaced size channels of the particle sizer.

### Bulk sediment δ^15^N measurements (previously published) and *z*-score calculations

The Site U1342 bulk sediment δ^15^N (δ^15^N_bulk_) data used in this study are from Knudson and Ravelo (*45*). In general, the δ^15^N_bulk_ record reflects changes in nitrate utilization (i.e., the fraction of the surface nitrate pool consumed by phytoplankton) because phytoplankton preferentially use ^14^N nitrate over ^15^N nitrate and carry this low-δ^15^N signature to the sediments as organic matter is exported (*57*). Thus, under low-nitrate utilization conditions, δ^15^N_bulk_ is low, and under high-nitrate utilization conditions, the δ^15^N_bulk_ value approaches the δ^15^N value of the source nitrate (δ^15^N_nitrate_) (*57*), which is ~5.5 to 8‰ in the surface and ~5 to 6‰ in the subsurface Bering Sea today (*58*). δ^15^N_bulk_ is also affected by changes in the δ^15^N values of δ^15^N_nitrate_, which can be altered by changing water masses with different δ^15^N_nitrate_ values and/or water column denitrification, which raises the δ^15^N_nitrate_ (*57*). The shallow, highly productive location of Site U1342 is preferable for recording δ^15^N_bulk_, as it is optimally located to avoid the isotopic effects of diagenetic alteration, because sediments deposited in high-productivity regions reflect the δ^15^N of the falling particles (*59*), and shallow/intermediate depth sites are less likely to undergo diagenesis compared to open ocean sites (*60*). Furthermore, the location of Site U1342 on the topographic high of Bowers Ridge ensures that deposited organic matter is of marine origin.

Knudson and Ravelo (*45*) calculated an orbital-scale record of nitrate utilization (Δδ^15^N) by subtracting a δ^15^N_bulk_ record representing background nitrate [ODP Site 1012 from Liu *et al.* (*61*)] from the U1342 δ^15^N_bulk_ record. However, the resolution of the Site 1012 δ^15^N_bulk_ record is not as high as the Site U1342 record (which has an average resolution of ~1 ka). Because of this discrepancy in resolution, the nitrate utilization (Δδ^15^N) record from Knudson and Ravelo (*45*) may not resolve millennial-scale changes, such as those characterizing laminated events. Therefore, in this study, we focus on the high-resolution changes in δ^15^N_bulk_ to characterize brief laminated intervals, keeping in mind that this measurement potentially reflects both changes in source δ^15^N values and in nitrate utilization.

To determine which laminated intervals were associated with statistically high values of δ^15^N_bulk_ relative to the mean, *z* scores were calculated as (χ − x*_t_*)/σ*_t_*, where χ is the value of the U1342 δ^15^N_bulk_ data that were detrended and normalized, x is the running mean over a 100-ka moving window at time *t*, and σ is the SD of the χ values over a 100-ka moving window at time *t*. Thus, positive *z* scores indicate points where the δ^15^N_bulk_ data are greater than the running mean. *Z* scores of 1.645 and greater indicate significantly high δ^15^N_bulk_ values at the *P* < 0.05 level. However, we note that because of the relatively wide temporal window used for the moving averages in the *z*-score calculations, some brief laminated intervals may have localized δ^15^N_bulk_ peaks that may not yield significant *z* scores although the δ^15^N_bulk_ peaks likely reflect true changes in environmental conditions.

### Statistical analyses with Monte Carlo hypothesis tests

The means of several Site U1342 records were assessed for statistically significant differences between laminated and nonlaminated intervals. These records included several productivity indicators (wt% N, Si/Ti ratios, GRA density, and NGR), as well as δ^15^N_bulk_ *z* scores, NPIW ventilation proxy (Site U1342 benthic δ^18^O minus Site 849 benthic δ^18^O, expressed as ∆δ^18^O) (*36*), and Fe/Ti and Fe/S ratios. All variables tested exhibit autocorrelation (i.e., data points within the time series were not statistically independent from the previous measurement), making basic methods of comparing means and proportions (e.g., two-sample *t* tests, chi-square tests of association) inappropriate. Moreover, because measurements were unevenly spaced through time, and because much longer laminated intervals occurred in the older half of the record, classical time series techniques are inappropriate. Thus, randomization-based hypothesis tests were programmed in R version 3.4.1 (*62*) to account for autocorrelation and unevenly spaced measurements. All code written to perform these analyses may be found at the following open source repository: https://github.com/knudson1/Bering.

For each test, the test statistic was defined as the sample mean during laminated intervals minus the sample mean during nonlaminated intervals; one test statistic was calculated from the original U1342 data, and one test statistic was calculated for each of the 10,000 simulated, randomized datasets. To prepare for the simulation, the empirical probabilities of switching states (from laminated to nonlaminated and vice versa) were calculated for consecutive measurements using the original Site U1342 data. For each of 10,000 iterations, a dataset was simulated by randomly designating each measurement “laminated” or “nonlaminated” using a Markov chain with the empirical transition probabilities, and the simulated dataset’s test statistic was calculated. The test statistic of the original data was then compared to the simulated test statistics: the *P* value was calculated using the proportion of simulated test statistics as extreme as or more extreme than the test statistic of the original data.

Two randomization tests were also conducted to determine whether laminated intervals were unusually likely or unlikely to occur during “glacial” and “interglacial” climates, compared to whether there was no association between laminated intervals and climate. The original test statistic was calculated as the proportion of laminated samples with a glacial U1342 benthic δ^18^O measurement (as defined previously). For each of 10,000 iterations, each δ^18^O measurement was randomly assigned as either laminated or nonlaminated using a Markov chain with the empirical transition probabilities, and the simulated test statistic (the proportion of laminated samples with a glacial value) was calculated. A significant association between laminations and climate is indicated if the test statistic of the original data is unusual compared to the test statistics of the simulated data (*P* ≤ 0.05): the *P* value was calculated using the proportion of simulated test statistics that are as extreme as or more extreme than the test statistic of the original data. The same test was repeated for interglacial measurements.

A final randomization test was conducted to determine whether the presence of laminated sediments was linked to global RSL. RSL from 0 to 514 ka ago was assigned from Rohling *et al.* (*63*) and from 515 to 1200 ka ago was assigned from Sosdian and Rosenthal (*64*). For this test, the test statistic was defined as the mean RSL during laminations. For each of 10,000 iterations, each RSL measurement was randomly assigned as either laminated or nonlaminated using a Markov chain with the empirical transition probabilities, and the simulated test statistic (the mean RSL during laminations) was calculated. A significant association between laminated sediments and RSL is indicated if the test statistic of the original data is unusual compared to the test statistics of the simulated data (*P* ≤ 0.05): The *P* value was calculated using the proportion of simulated test statistics that are as extreme as or more extreme than the test statistic of the original data.

## SUPPLEMENTARY MATERIALS

Supplementary material for this article is available at http://advances.sciencemag.org/cgi/content/full/7/23/eabg2906/DC1
